# Multi-modal co-learning with attention mechanism for head and neck tumor segmentation on ^18^FDG PET-CT

**DOI:** 10.1186/s40658-024-00670-y

**Published:** 2024-07-25

**Authors:** Min Jeong Cho, Donghwi Hwang, Si Young Yie, Jae Sung Lee

**Affiliations:** 1 Interdisciplinary Program in Bioengineering, Seoul National University College of Engineering, Seoul, 03080 South Korea; 2 Department of Nuclear Medicine, Seoul National University College of Medicine, 103 Daehak-ro, Jongno-gu, Seoul, 03080 South Korea; 3 Integrated Major in Innovative Medical Science, Seoul National Graduate School, Seoul, South Korea; 4 Department of Biomedical Sciences, Seoul National University College of Medicine, Seoul, 03080 South Korea; 5 Brightonix Imaging Inc, Seoul, 04782 South Korea

**Keywords:** PET/CT, Multi-modal image segmentation, Head and neck tumor segmentation, Attention, Co-learning

## Abstract

**Purpose:**

Effective radiation therapy requires accurate segmentation of head and neck cancer, one of the most common types of cancer. With the advancement of deep learning, people have come up with various methods that use positron emission tomography-computed tomography to get complementary information. However, these approaches are computationally expensive because of the separation of feature extraction and fusion functions and do not make use of the high sensitivity of PET. We propose a new deep learning-based approach to alleviate these challenges.

**Methods:**

We proposed a tumor region attention module that fully exploits the high sensitivity of PET and designed a network that learns the correlation between the PET and CT features using squeeze-and-excitation normalization (SE Norm) without separating the feature extraction and fusion functions. In addition, we introduce multi-scale context fusion, which exploits contextual information from different scales.

**Results:**

The HECKTOR challenge 2021 dataset was used for training and testing. The proposed model outperformed the state-of-the-art models for medical image segmentation; in particular, the dice similarity coefficient increased by 8.78% compared to U-net.

**Conclusion:**

The proposed network segmented the complex shape of the tumor better than the state-of-the-art medical image segmentation methods, accurately distinguishing between tumor and non-tumor regions.

**Supplementary Information:**

The online version contains supplementary material available at 10.1186/s40658-024-00670-y.

## Introduction

Head and neck (H&N) cancer is one of the most common cancers and the eighth most common cause of cancer-related deaths [1, 2]. Radiation therapy cannot be performed successfully without accurate segmentation of head and neck tumors. Traditionally, radiation oncologists, medical physicists, and radiologists have manually delineated the target tumors. However, manual drawing is laborious, time-consuming, and subject to high intra- and inter-operator variabilities [3]. Therefore, automatic segmentation methods for H&N oncology are required in clinical practice and research [4].

Computed tomography (CT) images are widely used in clinical practice owing to their excellent spatial resolution and ability to provide detailed anatomical information. However, the CT intensity of H&N tumors is similar to that of adjacent tissues, which makes H&N tumor segmentation more difficult [5]. On the other hand, [^18^F] fluorodeoxyglucose positron emission tomography (PET) is a powerful imaging technique that can visualize metabolically active tissues, such as cancer cells [6]. Therefore, hybrid positron emission tomography-computed tomography (PET-CT) plays an important role in the diagnosis and management of H&N cancer [7, 8].

With the advancement of deep learning, various methods that use complementary information from PET-CT have been proposed. Xu et al. [9] proposed the use of cascaded V-nets for 3D volume segmentation in PET-CT imaging. In this method, the first V-Net generates a binary mask for the target object, and the mask is fed to the second V-Net along with the PET-CT images. Moreover, another method of combining features extracted from the PET and CT images via two parallel encoder networks has been proposed [10–13]. However, this method ignores the interaction of information between the two modalities during feature extraction. Xue et al. [6] achieved feature interaction across multi-modal channels by sharing features during downsampling. However, these approaches are computationally expensive because of the separation of the extraction and fusion functions and do not take full advantage of the high sensitivity of PET. In contrast, Fu et al. [14] exploited the high sensitivity of PET without co-learning PET and CT.

The main objective of this study was to achieve simple yet effective feature co-learning while fully exploiting the high sensitivity of PET. For the co-learning of multi-modality, we designed a network that learns the correlation between PET and CT features through squeeze-and-excitation normalization (SE Norm) [15] without separating the feature extraction and fusion functions. Moreover, we propose the use of a tumor region attention module (TRAM), which allows our method to fully exploit the high sensitivity of PET.

**Fig. 1:**
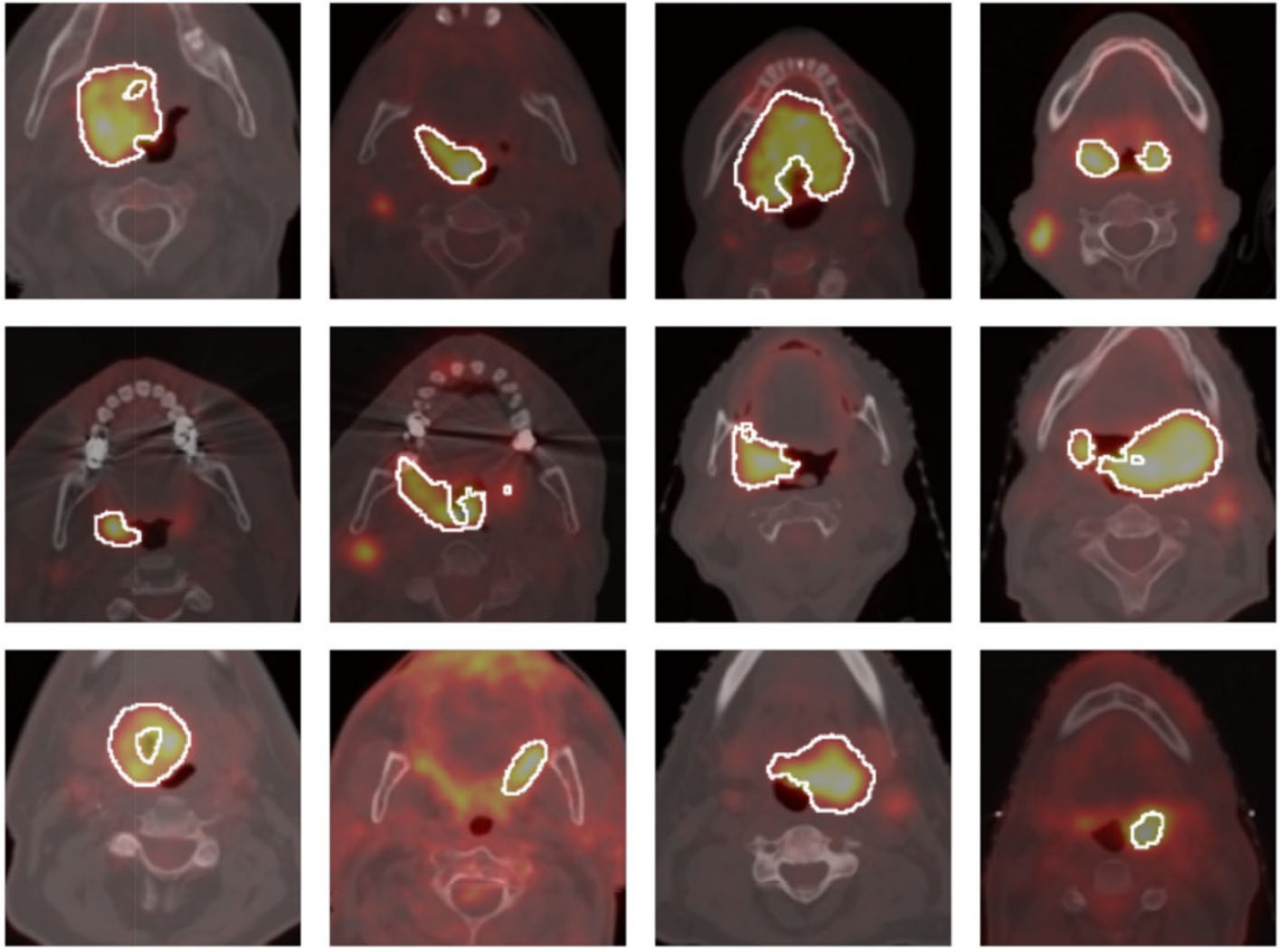
Various shape of head and neck tumors

A major challenge in H&N tumor segmentation is that, unlike normal organs in the body, H&N tumors have a variety of shapes and scales. For example, as shown in Fig. 1, the shape of the tumor is irregular, causing poor generalization. Therefore, to achieve strong generalization capability, it is essential to train the network by extracting contextual information from different environments. To this end, we introduce multiscale context fusion (MSCF), which exploits contextual information from different scales.

## Materials and methods

### Materials

In this study, we used 224 FDG PET-CT scans of patients with head and neck tumors located in the oropharynx region, which were provided in the MICCAI HECKTOR 2021 challenge [5, 16]. The label is a CT resolution-based 3D binary description of the primary gross tumor volume (GTVt), 1 for the tumor mask, and 0 for the background. The dataset was collected from five centers (HGJ, HMR, CHUM, CHUS, and CHUP). The voxel size and resolution of the original images differed because they were acquired from different institutions.

To achieve a better and more consistent performance of the proposed network, the PET and CT images were preprocessed. First, we resampled the images to 1 $\:\times\:$ 1 $\:\times\:$ 1 mm^3^ to obtain the same voxel size. For resampling, trilinear interpolation was used for the PET and CT images, and nearest-neighbor interpolation was used for label images. The dataset we utilized provides bounding box information necessary for locating the tumor region. The dimensions of these bounding boxes are 144 $\:\times\:$ 144 $\:\times\:$ 144 mm^3^. Based on this information, the images were cropped to a size of 144 $\:\times\:$ 144 $\:\times\:$ 144 mm^3^. The intensities of CT images were clipped within the range of [-1024, 1024] Hounsfield units. Subsequently, these values were mapped to the range of [-1, 1]. The Z-score normalization was performed for PET image intensities.

### Implementation details

We trained the network for 100 epochs using Python and Pytorch on a single GeForce RTX 3090 graphics processing unit. The same settings and hyperparameters were applied in all the experiments to compare the proposed model with the state-of-the-art models. The batch size was set as 1. The initial learning rate for the Adam optimizer was set to 0.001. The scheduler is a cosine annealing scheduler.

The data were split at 80:10:10 ratios for the training, validation, and test, respectively. Although data were generated from five centers, they were randomly shuffled and split without taking this into account. During training, random flips and rotations were applied for data augmentation.

Network models were comparatively evaluated using the Dice similarity coefficient (DSC), Hausdorff distance (HD), precision, and recall. The main matrices for comparison, DSC and HD, are defined as follows: (1) Dice similarity coefficient (DSC): This shows the extent of overlap between the prediction and the ground truth, ranging between 0 and 1. The DSC closer to 1 indicates that the prediction and ground truth are similar, which is defined as

$$\:DSC\left({V}_{P},\:{V}_{G}\right)=\frac{2\left|{V}_{P}\:\cap\:{V}_{G}\right|}{\left|{V}_{P}\right|+\:\left|{V}_{G}\right|},$$

where $\:{V}_{P}$ denotes the prediction for the H&N tumor and $\:{V}_{G}$ denotes the ground truth.

(2) Hausdorff distance (HD): It calculates the maximum distance between two segmentation surfaces and indicates the closeness between the predicted and ground truth boundaries, which is defined as

$$\:{D}_{H}\left(P,\:G\right)=\text{max}\left\{\underset{p\:\in\:\:P}{\text{s}\text{u}\text{p}}\:\underset{g\:\in\:\:G}{\text{i}\text{n}\text{f}\text{p}}d\left(p,\:g\right),\:\underset{g\:\in\:\:G}{\text{s}\text{u}\text{p}}\:\underset{p\:\in\:\:P}{\text{i}\text{n}\text{f}\text{p}}d\left(p,\:g\right)\right\},$$

where $\:d(p,g)$ is the Euclidean distance between points $\:p$ and $\:g$.

### Overall networks

**Fig. 2:**
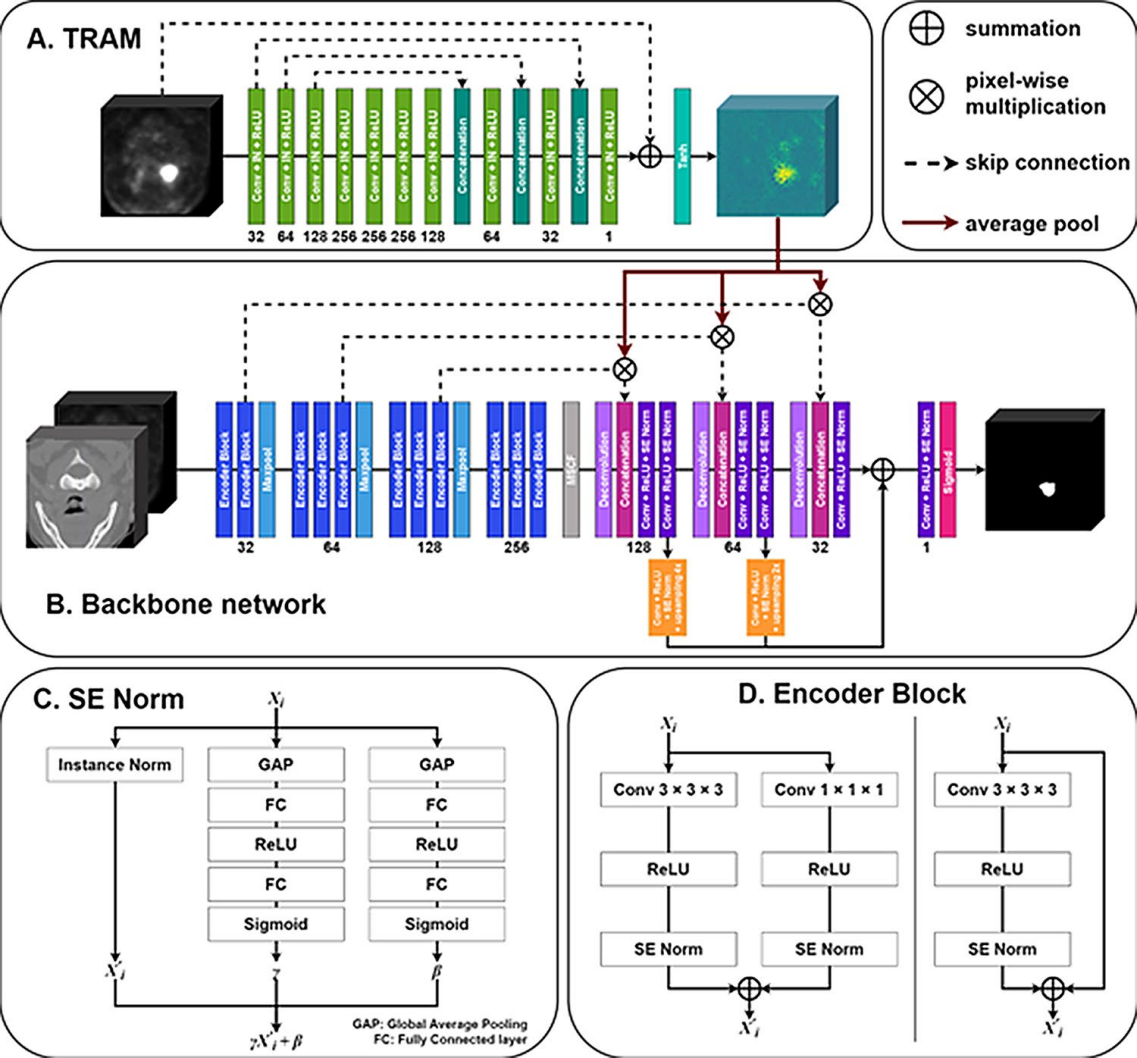
Overall architecture of our proposed model. PET and CT concatenated as two channels are input to the backbone network, and only PET is input to the TRAM. The numbers below each block indicate the number of channels. D represents the detailed block of the encoder block, and there are two types: the left one is used when the number of feature channels of input and output are different, and the right one is employed when the number of feature channels of input and output are the same

Figure 2 shows a schematic of the proposed network based on U-Net [17] as the backbone. The proposed network consists of three main components: TRAM, MSCF, and SE Norm. To extract features from the PET images, TRAM is separated from the backbone network, and the output of the TRAM is multiplied by the feature maps from the encoder of the backbone and inserted into the decoder. Both the MSCF and SE Norm are inserted into the backbone network. The MSCF, which exploits the different contextual information of high-level feature maps, is placed between the encoder and decoder. In deep learning, most people apply rectified linear unit (ReLU) activation function and normalization sequentially after the convolutional layer and this process are used as one block. The proposed model uses the SE Norm as a normalization of this block.

#### Feature encoder

We used two types of blocks in the encoder of the backbone U-Net architecture. One was used to make the channel sizes of the input and output different. The second is used when the channel sizes of the input and output are the same. Both are composed of a 3 $\:\times\:$ 3 $\:\times\:$ 3 convolution layer, ReLU activation function, SE Norm, and a residual layer. The difference is that only the residual layer in the former block contains a 1$\:\times\:$1$\:\times\:$1 convolution layer, a ReLU activation function, and SE Norm.

#### Tumor region attention module

The use of the TRAM subnetwork is proposed considering the higher sensitivity of PET images. From the PET image $\:\varvec{P}\in\:{\mathbb{R}}^{1\:\times\:\:D\:\times\:\:H\:\times\:\:W}$, TRAM generates a tumor attention map $\:\varvec{A}\in\:{\mathbb{R}}^{1\:\times\:\:D\:\times\:\:H\:\times\:\:W\:}$ to guide the tumor localization. A tumor attention map is incorporated into the segmentation backbone. The TRAM architecture is based on a modified U-Net consisting of a series of layers, normalizations, and activation functions. For each layer in the contracting path, a 3 $\:\times\:$ 3 $\:\times\:$ 3 convolution layer with a stride of two reduces the input size by half instead of 2 $\:\times\:$ 2 $\:\times\:$ 2 pooling layers, which is followed by batch normalization and ReLU. In the expanding path, the upsampling layer doubled the image dimensions by applying trilinear interpolation, and the number of channels was reduced by half through a 3 $\:\times\:$ 3 $\:\times\:$ 3 convolution layer. We applied dropout to concatenation in the decoder to reduce overfitting and add stochasticity to the network. The dropout rate is 0.5. We used the tanh activation function as the last layer to highlight meaningful features, such as tumors, and suppress less useful ones. The details of TRAM are shown in Fig. 2A.

#### Squeeze-and-excitation normalization

H&N tumors on PET images usually show high FDG uptake. However, some non-tumor regions also have high physiological FDG uptake. Therefore, the classification of tumors and non-tumors based solely on SUV values in PET images can be erroneous. Therefore, our network used SE Norm which enhances inter-channel relationships within the combined CT and PET feature maps to effectively blend anatomical information from CT images and metabolic information from PET images (Fig. 2C).

This module is based on the squeeze-and-excitation block, which considers the interdependencies between the channels. The inputs to the backbone network were two channels: concatenated CT and PET images. Subsequently, SE Norm was performed on each layer, and three processes were performed as follows. First, the input features were normalized by applying instance normalization. Second, because this process is performed at each channel, the output of instance normalization does not consider the information correlation between channels; therefore, to solve this problem, we generated a pair of parameters $\:{\gamma\:}_{i},\:\:{\beta\:}_{i}\:$from input features through SE blocks. Finally, these normalized values were scaled and shifted by $\:{\gamma\:}_{i}$ and$\:{\:\beta\:}_{i}$.

$$\:{y}_{i}={\gamma\:}_{i}{x}_{i}^{{\prime\:}}+{\:\beta\:}_{i}$$

where $\:{x}_{i}^{{\prime\:}}\:$is the output of the instance normalization and $\:{y}_{i}$ is the final output of the SE Norm.

#### Multi-scale context fusion

**Fig. 3:**
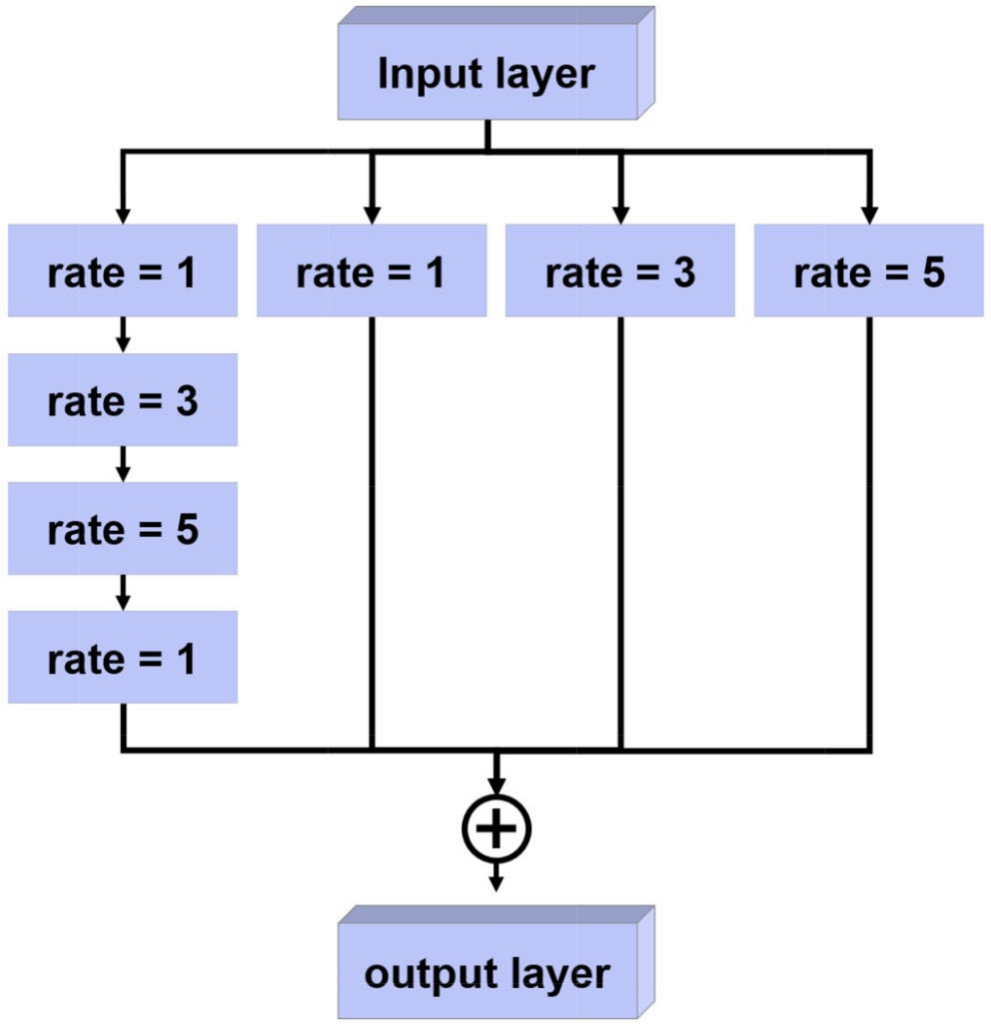
Structure of the proposed multi-scale context fusion. The term “rate” means the dilation rate of the atrous convolution. Also, the fusion of the four features into one is achieved by summation

To strengthen the generalization ability of the network for diverse H&N tumor shapes, we designed an MSCF that fuses contextual information on various scales (Fig. 3) [18]. By inserting the MSCF between the encoder and decoder, we can address the issue of spatial information loss caused by consecutive pooling operations in the encoder. In this module, we extract four features at different scales by utilizing atrous convolution with different kernel sizes, which expands the receptive field of the kernels while reducing the computational burden caused by increasing the kernel size [19]. The receptive field sizes used for atrous convolution were 3,7,11 and 19, and the kernel sizes for each atrous convolution are shown in Fig. 3. The contextual information is then fused by combining the four features into one.

#### Feature decoder

To achieve accurate segmentation results with high resolution, the tumor attention generated by the TRAM and the SE Norm were also employed in the decoder path. The skip connection that delivers the features from the encoder is multiplied by the attention map to guide the tumor localization. Each block consists of a 3 $\:\times\:$ 3 $\:\times\:$ 3 convolution, ReLU, and SE Norm (Fig. 2, purple block). We also added three upsampling paths in the decoder to transmit low-resolution features, which were composed of 1 $\:\times\:$ 1 $\:\times\:$ 1 convolution to minimize the number of channels and trilinear interpolation to enlarge the feature map (Fig. 2, red block). Finally, to yield a segmentation probability map, the sigmoid function was applied.

### Loss function

In deep learning, segmentation is usually considered as pixel-wise classification; therefore, the cross-entropy loss function is most commonly used for segmentation tasks. However, because the range of tumor size on PET-CT is considerably wide, this loss of function would not be ideal in this study. Therefore, we used the soft Dice loss function instead of the cross-entropy loss function. The Dice similarity coefficient is the most common metric for calculating the similarity between two images in computer vision, especially for medical images. The soft dice loss is defined as:

$$\:{L}_{Soft\:Dice\:loss}\left(y,\:\widehat{y}\right)=\:1-\:\frac{2{\sum\:}_{i}^{N}{y}_{i}{\widehat{y}}_{i}\:+\:\epsilon\:}{{\sum\:}_{i}^{N}{y}_{i}^{2}+\:{\sum\:}_{i}^{N}{\widehat{y}}_{i}^{2}\:+\:\epsilon\:}\:$$.

Our method is considered as a single integrated model and trained end-to-end using soft dice loss without applying additional constraints.

## Results

### Ablation experiments

Different combinations of strategies were compared to investigate the effect of incorporating the three proposed strategies (TRAM, SE Norm, and MSCF) to improve H&N tumor segmentation performance (Table 1). Adoption of TRAM, SE Norm, or MSCF resulted in better performance than the native U-Net. Among the combinations of the two strategies, the combination of TRAM and SE Norm exhibited the best performance. The proposed model with all three proposed strategies outperformed the other combinations. The DSC of the proposed model incorporating all three techniques was 8.78% greater than the DSC of the U-Net. In addition, the model reduced the number of false positives, as evidenced by its higher precision than U-Net.

**Table 1:** Quantitative evaluation (the mean of DSC, HD, Precision, and Recall) of combination with partial and whole modules for head and neck tumor segmentation. All three modules were inserted into the proposed. Instance normalization was applied for the networks not using SE Norm

	DSC	HD	Precision	Recall
U-Net	0.672$\:\pm\:$0.308	17.832$\:\pm\:$21.422	0.722$\:\pm\:$0.323	0.702$\:\pm\:$0.332
TRAM	0.686$\:\pm\:$0.280	17.041$\:\pm\:$17.794	0.704$\:\pm\:$0.272	0.751$\:\pm\:$0.309
MSCF	0.693$\:\pm\:$0.265	18.506$\:\pm\:$17.998	0.724$\:\pm\:$0.277	0.736$\:\pm\:$0.300
SE Norm	0.718$\:\pm\:$0.241	14.421$\:\pm\:$17.160	0.719$\:\pm\:$0.268	0.766$\:\pm\:$0.250
TRAM+MSCF	0.700$\:\pm\:$0.248	17.618$\:\pm\:$18.278	0.740$\:\pm\:$0.290	0.718$\:\pm\:$0.257
TRAM+SE Norm	0.724$\:\pm\:$0.249	18.501$\:\pm\:$21.504	0.742$\:\pm\:$0.277	0.761$\:\pm\:$0.242
MSCF+SE Norm	0.721$\:\pm\:$0.274	**11.615** $\:\pm\:$ **11.014**	0.743$\:\pm\:$0.297	0.749$\:\pm\:$0.290
Proposed	**0.731** $\:\pm\:$ **0.228**	13.393$\:\pm\:$15.813	**0.756** $\:\pm\:$ **0.257**	**0.768** $\:\pm\:$ **0.236**

**Fig. 4:**
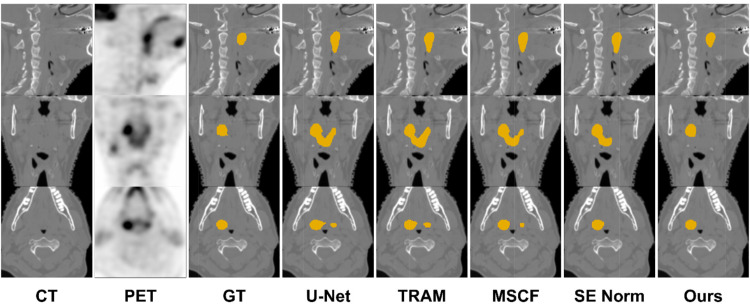
Segmentation result of a representative patient using U-Net only, TRAM, MSCF, SE Norm, and proposed (TRAM + MSCF + SE Norm)

**Fig. 5:**
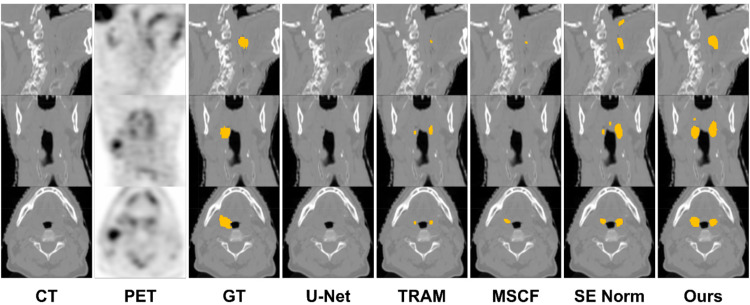
Segmentation result of another representative patient using U-Net only, TRAM, MSCF, SE Norm, and proposed (TRAM + MSCF + SE Norm)

Figure 4 shows an example of the segmentation results using a native U-net, a single improvement strategy (TRAM, SE Norm, or MSCA), and a combination of three strategies (proposed). In this case, only the proposed method distinguished the H&N tumor from other hot-uptake nontumor regions. Figure 5 shows that TRAM, SE Norm, and MSCF outperform native U-Net in tumor delineation.

### Comparison with the state-of-the-art

**Fig. 6:**
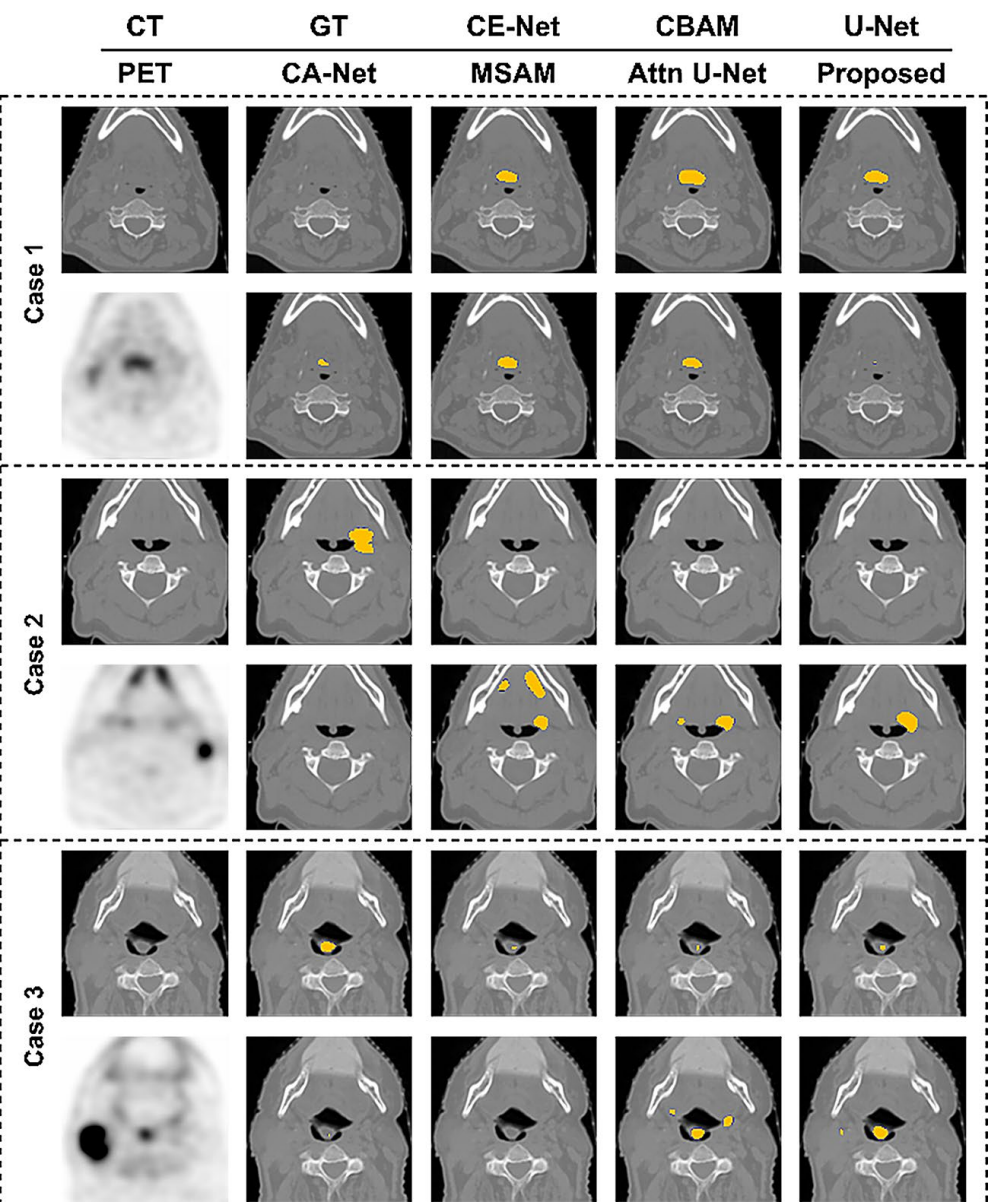
Comparison of the proposed model and the state-of-the-art methods for medical image segmentation. CBAM: U-net with convolutional block attention module (CBAM)

Various U-Net-like models have been proposed for medical image segmentation, such as CE-Net [20], CBAM [21], CA-Net [22], MSAM [14], and Attention U-Net [23]. CE-Net combines U-Net with a novel context extractor. By placing a novel context extractor between the feature encoder and decoder in its architecture, CE-Net can avoid information loss due to successive pooling and convolution operations and highlight meaningful high-level features [20]. The network also learns variational context information using atrous convolution (See Fig. 6).

Attention U-Net, CBAM, and CA-Net use attention modules that focus on the correlation between features. In these methods, co-learning can be achieved by feeding PET and CT into the networks as two channels and focusing on the correlation between features. Attention U-Net has proposed an attention gate integrated into U-Net while minimizing computation overhead [23]. CBAM is a simple yet effective module. By applying this module to the convolution output in each block, CBAM highlights important characteristics and learns correlations in two dimensions: channels and spatial axes [21]. CA-Net uses multiple attention in convolutional neural networks to allow networks to learn spatial position, channel, and scale simultaneously [22]. To take full advantage of PET’s high sensitivity, MSAM trains a sub-network that focuses on notable regions of the PET images and a main network that provides segmentation results [14].

The proposed method focuses on the target region through co-learning, fully exploits the sensitivity of PET, and learns the variations in the shape and size of the tumors by capturing contextual information. As shown in Table 2, the proposed method outperformed the state-of-the-art models mentioned above.

**Table 2:** Quantitative comparison of the proposed model and the state-of-the-art. CBAM named the model to which CBAM was applied just before each pooling in U-Net. Except for the proposed method, other networks applied instance normalization

	DSC	HD	Precision	Recall
CE-Net	0.664$\:\pm\:$0.314	20.483$\:\pm\:$21.176	0.691$\:\pm\:$0.315	0.728$\:\pm\:$0.346
CBAM	0.671$\:\pm\:$0.298	20.961$\:\pm\:$22.387	0.709$\:\pm\:$0.274	0.755$\:\pm\:$0.329
U-Net	0.672$\:\pm\:$0.308	17.832$\:\pm\:$21.422	0.722$\:\pm\:$0.323	0.702$\:\pm\:$0.332
CA-Net	0.672$\:\pm\:$0.336	**13.288** $\:\pm\:$ **17.229**	0.737$\:\pm\:$0.216	0.683$\:\pm\:$0.353
MSAM	0.703$\:\pm\:$0.291	14.384$\:\pm\:$18.470	0.742$\:\pm\:$0.287	0.742$\:\pm\:$0.302
Attention U-Net	0.718$\:\pm\:$0.250	15.538$\:\pm\:$17.604	0.733$\:\pm\:$0.275	0.764$\:\pm\:$0.252
**Proposed**	**0.731** $\:\pm\:$ **0.228**	13.393$\:\pm\:$15.813	**0.756** $\:\pm\:$ **0.257**	**0.768** $\:\pm\:$ **0.236**

### Assessment of module effectiveness through visual analysis

Our research objective was to achieve simple yet effective feature co-learning by fully exploiting the high sensitivity of PET imaging. To this end, we employed two modules: TRAM and SE Norm. To understand how each module functions, we conducted further analyses.

**Fig. 7:**
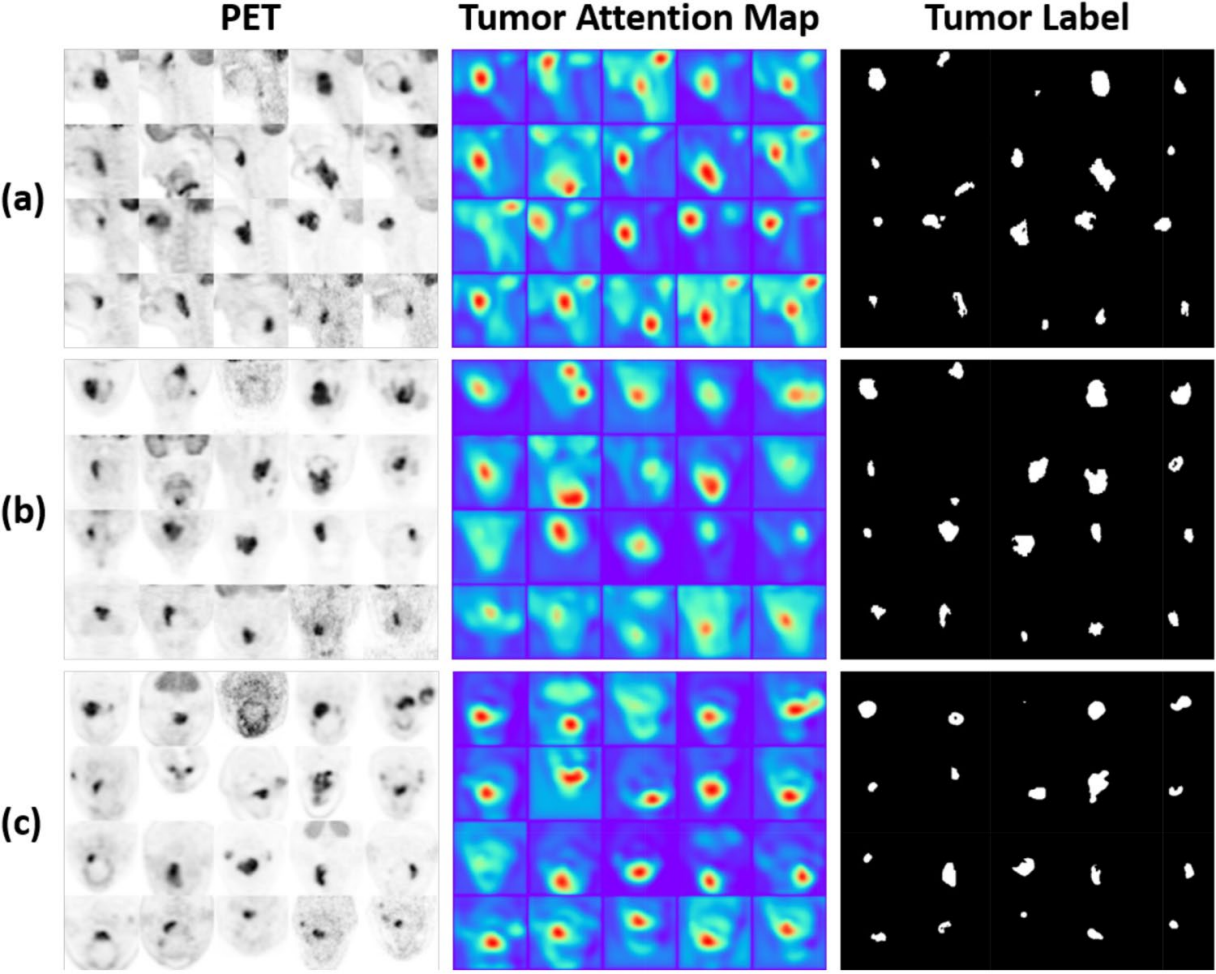
Visualization of tumor attention map extracted from TRAM; (**a**) Sagittal (**b**) Coronal (**c**) Axial

**Fig. 8:**
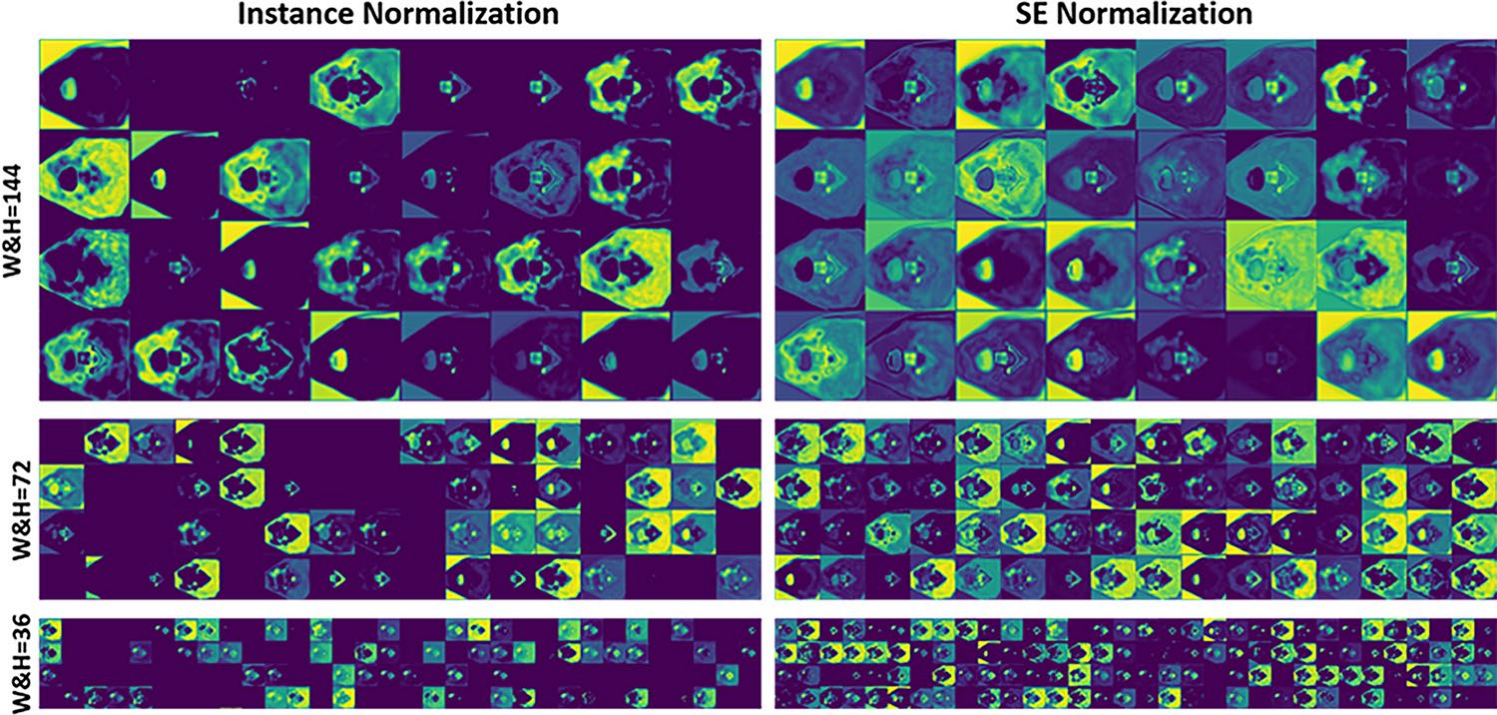
Feature maps from an encoder with the application of instance normalization and SE normalization

Figure 7 displays the Tumor Attention Map generated by TRAM. This map effectively highlights areas of high uptake within PET images, indicating TRAM’s ability to focus attention on regions of interest. In essence, this suggests TRAM’s role in aiding the network in distinguishing more clearly between normal and abnormal tissues. Figure 8 visualizes the feature maps extracted from the encoder when SE Norm is applied versus when only instance normalization is used. A comparison of the two feature maps demonstrates that the use of SE Norm results in the preservation of more anatomical information. As depth of U-Net increases, feature maps that rely solely on instance normalization show a greater emergence of blank spaces. However, the application of SE Norm significantly mitigates this issue, showcasing its effectiveness in maintaining feature information at deeper network levels. These findings illustrate the pivotal role of SE Norm in enhancing feature co-learning within deep learning architectures.

## Discussion

In multi-modal medical image processing [24, 25], it is useful to combine image features because the information included in each image modality is unique and complementary. Utilizing attention modules focusing on target structures and co-learning the two features are useful ways to effectively combine the image features. Appropriate utilization of PET uptake information is also important if PET is available for segment tumors. Taking all these points into account, we developed a novel deep learning-based strategy to improve the performance of H&N tumor segmentation in PET-CT. The proposed network can more effectively complement PET and CT by co-learning multi-modal features with SE Norm and guiding tumor localization with TRAM.

Attention U-Net [23] and CBAM [21] increase segmentation performance by applying additional attention modules after the convolution operation. When this method is applied to PET/CT, the features of each image are first extracted through convolution, and the extracted features are fused by the additional attention modules to perform co-learning. However, the separate extraction and fusion increases the computational complexity. In convolutional neural networks, the convolutional operation consists of a convolutional layer, an activation function, and a normalization layer. The normalization layers typically employ batch normalization or instance normalization. However, we have used SE Norm [15] as the normalization layer, which allows simultaneous feature extraction and co-learning of features. On the other hand, MSAM [14] executes learning that focuses on the high uptake of PET without any co-learning between PET and CT, which may misclassify some regions with high physiological PET uptake as tumors. In contrast to MSAM, TRAM utilizes the high sensitivity of PET and reduces false positives by combining the anatomical and physiological information of CT and PET. As shown in Table 1, the use of SE Norm with TRAM is more successful than without TRAM.

The objects of medical image segmentation, such as organs and tumors, are diverse. Normal organs usually have a similar shape, but tumors come in a variety of sizes, shapes, and locations. Therefore, networks for tumor segmentation require appropriate contextual information. CE-Net [20] devised a new context extractor and placed it between U-net encoder and decoder to learn about contextual information and highlight high-level meaningful features. In a similar way, we devised MSCF to be able to extract and learn contextual information at various scales using atrous convolution and added it to TRAM and SE Norm. Table 1 shows that when MSCF was employed with TRAM and SE Norm, DSC increased by 0.97%, which demonstrates the effectiveness of MSCF in improving the generalization ability.

We investigated the effects of combining three elements (TRAM, SE Norm, and MSCF). Learning with only one element performed worse than the two combinations, indicating that each element serves to compensate for the shortcomings of the other method. Among the two combinations, the TRAM and SE Norm combination showed the best performance. Similarly, combining all three boosted the performance improvement.

The outcomes of our study, compared with state-of-the-art (SOTA) methods, are presented in Table 2, which implies the following insights:

1. Contextual information: The proposed model showed better results than CE-Net with 10.1% increase in DSC and 7.09 mm decreased in HD. Our model also delineated the tumor boundary better than CE-Net, as shown in Case 3 in Fig. 6.
2. PET sensitivity: Compared to MSAM trained on the advantage of utilizing PET’s high sensitivity, our model performed better in all metrics, especially with a 3.98% increase in DSC. Figure 6 shows that false negatives and false positives of MSAM are higher than those of the proposed.
3. Co-learning: Among the methods using attention modules focusing on the correlation of features, Attention U-Net showed the highest performance and CBAM showed the lowest. Our proposed model achieved 1.8% and 8.94% higher DSC than Attention U-Net and CBAM, respectively. Case studies 2 and 3 in Fig. 6 show that Attention U-Net yields more false positives than the proposed model.

However, other mothods have a better Hausdorff Distance (HD) than the proposed method in Tables 1 and 2. TRAM is the cause for the results. In order to segment tumors more accurately, TRAM generates a map that pays attention to high uptake in PET, and with the help of that, our proposed method performs segmentation. In Fig. 7, we can see a visualization of the tumor attention map, which usually gives attention to a larger area than the actual size of the tumor. Therefore, this is the reason HD becomes larger than other networks.

As demonstrated in this study, co-learning the features of multi-modal images armed with an attention mechanism that utilizes images with higher sensitivity is an effective strategy for accurate H&N tumor segmentation in PET-CT. To the best of our knowledge, this is the first study to utilize the complementary information of two modalities while exploiting the high sensitivity of PET.

## Conclusions

We developed a method for H&N tumor segmentation in PET-CT using a deep neural network model that complements PET and CT more effectively by fully exploiting the high sensitivity of PET using SE Norm and guiding tumor localization using TRAM, and improved the generalization ability of the network model by utilizing contextual information with MSCF. The effectiveness of these three strategies in improving the H&N tumor segmentation was proven by comparing the performance of the proposed method with that of other state-of-the-art methods.

### Electronic supplementary material

Below is the link to the electronic supplementary material.

Supplementary Material 1

## Data Availability

All data generated or analyzed during this study are included in this published article.
