# Supplementary material for: Multi-modal co-learning with attention mechanism for head and neck tumor segmentation on 18FDG PET-CT
Source: EJNMMI Phys. 2024 Jul 25;11:67. doi: 10.1186/s40658-024-00670-y (PMC11272764; doi:10.1186/s40658-024-00670-y)
Supplement: Supplementary file 1 — Supplementary Material 1 [file 40658_2024_670_MOESM1_ESM.docx]

**The number of parameter**

1. **Table 1**

| **Method** | **Param #** |
| --- | --- |
| U-Net | 6419137 |
| TRAM | 12564776 |
| MSCF | 17167553 |
| SE Norm | 9602177 |
| TRAM + MSCF | 23313192 |
| TRAM + SE Nprm | 15747816 |
| MSCF +SE Norm | 20350593 |
| Proposed | 26496232 |

1. **Table 2**

| **Method** | **Param #** |
| --- | --- |
| CE-Net | 16285271 |
| CBAM | 6477013 |
| U-Net | 6419137 |
| CA-Net | 6175919 |
| MSAM | 12836546 |
| Attention U-Net | 6441897 |
| Proposed | 26496232 |

**Visualization of feature maps**


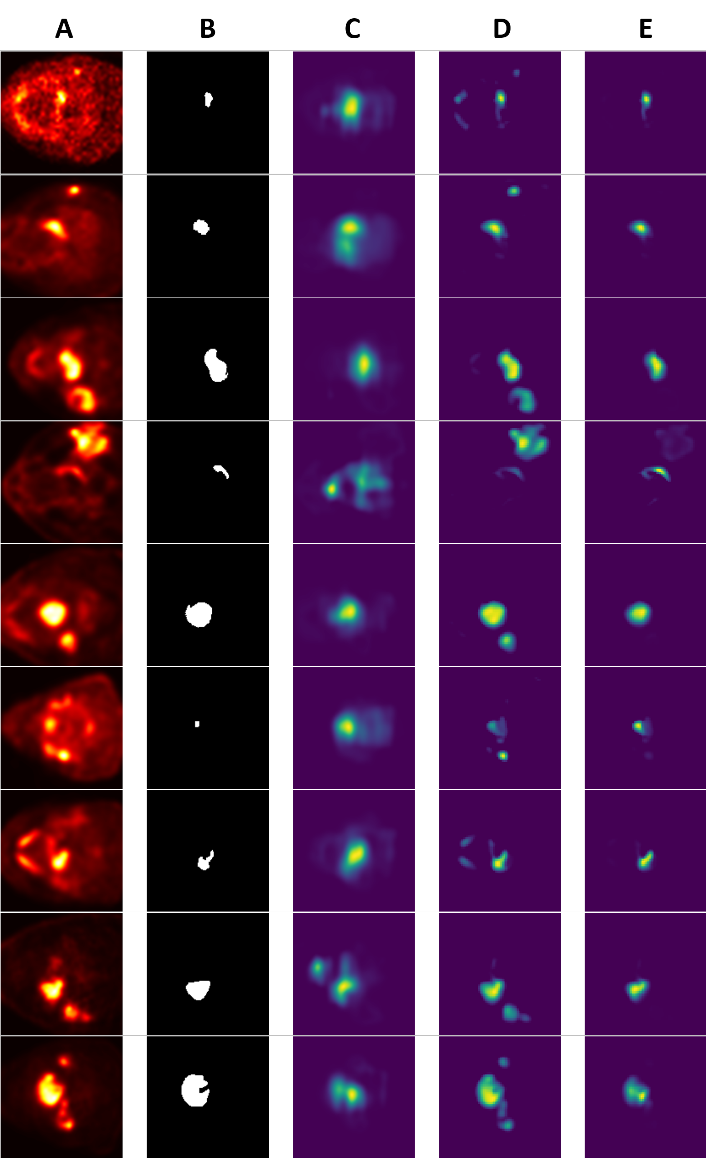


(A: Positron Emission Tomography (PET), B: Tumor, C: Tumor attention map, D: Feature map of the backbone network, E: Multiplication of the feature map of the backbone network and the tumor attention map)
